# Concurrent validity of the short version of Montreal Cognitive Assessment (MoCA) for patients with stroke

**DOI:** 10.1038/s41598-021-86615-2

**Published:** 2021-03-30

**Authors:** Yali Feng, Jiaqi Zhang, Yi Zhou, Bo Chen, Ying Yin

**Affiliations:** 1 Department of Rehabilitation Medicine, The Second Affiliated Hospital of Chongqing Medical University, Chongqing, China; 2 Department of Rehabilitation Sciences, The Hong Kong Polytechnic University, Hung Hom, Hong Kong SAR China; 3 Department of Rehabilitation Medicine, The First Affiliated Hospital of Southwest Medical University, Luzhou, Sichuan China

**Keywords:** Geriatrics, Cerebrovascular disorders, Dementia

## Abstract

The aim of the present study was to examine the concurrent validity of 2 Chinese versions of the short version of the Montreal Cognitive Assessment (MoCA) in patients with stroke, i.e., MoCA 5-minute protocol and National Institute for Neurological Disorders and Stroke and Canadian Stroke Network (NINDS-CSN) 5-minute Protocol. A total of 54 patients and 27 healthy controls were enrolled in this study. In this study, the Neurobehavioural Cognitive Status Examination (NCSE) was used as an external criterion of cognitive impairment. We found that the 5-min protocol did not differ from the MoCA in differentiating patients with cognitive impairments from those without (area under the receiver operating characteristic curve, AUC, of 0.948 for the MoCA 5-min protocol *v.s.* 0.984 for MoCA, *P* = 0.097). These three assessments demonstrated equal performance in differentiating patients with stroke from controls. The Chinese version of the MoCA 5-min protocol can be used as a valid screening for patients with stroke.

## Introduction

The Montreal Cognitive Assessment (MoCA) was developed by Nasreddine et al. in 1996, and was initially used and validated to detect mild cognitive impairment (MCI) and mild forms of Alzheimer’s disease^1^. Recent evidence has demonstrated that the MoCA is, at the present moment, the best screening tool for MCI (pooled sensitivity, 89%; pooled specificity, 75%)^2^, and that it is also an excellent screening tool for post-stroke cognitive impairment (pooled sensitivity, 81%; pooled specificity, 79%)^3^. Although MoCA is primarily designed as a cognitive screening tool, it is still relatively time consuming (taking approx. 15 min), with limited feasibility for use with patients with either low education levels, or with unstable conditions (e.g., frailty, severe attention deficits, and mind-wandering)^4^. In recent years, there has been an emerging interest in concentrating the most useful items from the MoCA into a similarly efficacious, but shorter version of MoCA. In 2006, the National Institute for Neurological Disorders and Stroke-Canadian Stroke Networkproposed a 5-minute protocol that can be adopted as a possible cognitive screening tool for patients with stroke, by extracting three items (i.e., orientation, memory and verbal fluency) from the MoCA. This assessment protocol is aimed to meet the requirement of busy clinics and also to allow for administration over the telephone, which is very promising and helpful for poststroke cognitive screening^5^.

In accordance with the recommendations of the NINDS-CSN, two short versions of MoCA have been developed to date^6,7^. Pendlebury et al. are the first group to validate a short version of MoCA (i.e., NINDS-CSN 5-min protocol) in patients with stroke or transient ischemic attack (TIA), reporting that NINDS 5-min protocol was overall less effective to the full MoCA in screening MCI among patients with stroke or TIA^6^. Another study which used the clinical diagnosis criteria of the American Heart Association/American Stroke Association for post-stroke dementia as the reference standard, found that the NINDS-CSN 5-min protocol has an acceptable discriminative power (sensitivity 82%, specificity 67%), and therefore can be a useful significant predictor of post-stroke dementia (odds ratio [OR], 6.32)^8^. A study by Dong et al. suggested that the NINDS-CSN 5-min protocol could predict the occurrence of late onset MCI (i.e., 1-year after stroke), though its predictive validity was slightly poorer than the full MoCA^9^. When compared to other validated cognitive screening tasks, such as the full MoCA^10^ as well as the Clinical Dementia Rating scale (CDR)^11^, the diagnosis accuracy of NINDS-CSN 5-min protocol was found to be high with balanced sensitivity and specificity. A second short version of MoCA was developed by Wong et al.^7^, which was later named as the MoCA 5-min protocol^4^. This protocol uses the same criteria (i.e., orientation, delayed recall and verbal fluency) from the full MoCA like the NINDS-CSN 5 min protocol, but with minor modifications in the assessment instruction and the grading system. To date, only four studies examining the Chinese version of the MoCA 5-min protocol have been published (2 in Hong Kong and 2 in Nanjing)^4,7,12,13^. This protocol has shown an acceptable level of reliability, which includes test–retest reliability and internal consistency^4,13^. We considered the concurrent validity of the MoCA 5-min protocol by first, its ability to detect cognitive impairments among patients with stroke^4,13^, and second, to distinguish patients with stroke from healthy controls^7,12^. The noted area under curve (AUC) of the MoCA 5-min protocol ranged from 0.76 to 0.86. Furthermore, one of these studies also found that the MoCA 5-min protocol is as effective as the full MoCA in differentiating stroke or TIA cohorts that have cognitive impairments (CDR > 0.5) from those without cognitive impairments (AUC: 0.78 *v.s.* 0.74,* P* > 0.05)^4^.

These short versions of MoCA that are based on the NINDS-CSN recommendation (i.e., the NINDS-CSN 5-min protocol by Pendlebury et al. and the MoCA 5-min protocol by Wong et al.) have gained wide traction in clinical practice and in the research of neurologists and neuropsychologists, but have not widely used by rehabilitation practitioners. To examine the effectiveness of the test, we will examine its concurrent validity, which is a measure of how well a new test compares to a well-established test, which is deemed as a fast way to validate the index test. Concurrent validity can be examined by investigating the correlation between the new test and the well-established test or testing the diagnostic accuracy in discriminating 2 known groups^14^. The objective of this study is to investigate the concurrent validity of these two short versions of MoCA, with reference to the Neurobehavioral Cognitive Status Examination (NCSE), which is one of the most widely used assessments for general cognition by neurological occupational therapists in China^15^. Concurrent validity was assessed using correlations and receiver operating characteristic (ROC)-analyses. We hypothesized that these two short versions of MoCA are highly correlated with the NCSE. We also explored whether the 2 short versions of MoCA could be alternatives to the full MoCA in screening of poststroke cognitive impairment. We hypothesized that the MoCA 5-min protocol performs similarly to full MoCA, and is more effective than the NINDS-CSN 5 min protocol in differentiating cognitively impaired from cognitively intact stroke patients as well as in differentiating patients with stroke from their age and education-matched healthy counterparts.

## Methods

Potential patients were prospectively recruited from two centers in Chongqing, a city in southwest China, from September 2018 to August 2019. Ethical approval was obtained from the Ethics Committee of the Second Affiliated Hospital, Chongqing Medical University before recruitment (Reference number: 2018-252). All participants provided their written informed consent before participation.

### Inclusion and exclusion criteria

The participants who met all of the following inclusion criteria were included: (1) patients diagnosed with unilateral hemispheric, ischemic or hemorrhagic stroke, with neuroimaging evidence from either computer tomography (CT) or magnetic resonance imaging (MRI); (2) stroke patients in the subacute phase (i.e., more than 1 month after stroke onset); (3) patients that were aged between 18 and 80 years; (4) Chinese (Mandarin)-speakers; and (5) patients who are able to communicate and to follow at least one-step commands.

The participants who met any of the following criteria were excluded: (1) patients with severe aphasia, swallowing disorders or other complications post-stroke that prevent them from successfully completing the assessments; (2) patients with any previously known psychiatric disorder or neurological disease excluding stroke; (3) patients who have been assessed by the MoCA or NCSE within the past 3 months and (4) those who refuse to participate in this study.

Cognitively normal healthy controls were enrolled from the relatives of the patients or staff in the hospital, with an inclusion criterion of: (1) age between 18 to 80 year; (2) no known history of neurological or psychiatric disorders; (3) no complaints of prior cognitive issues; (4) Chinese (Mandarin)-users and (5) agreement to participate in this study.

## Materials and procedures

### Montreal Cognitive Assessment (MoCA)

The MoCA Chinese (Mandarin) version-1 (from http://www.mocatest.org) was used in this study. The total score for the MoCA is 30 and it covers seven domains of cognition: visuospatial/executive functions (trail-making test: 1 point, copy tube: 1 point and clock drawing task: 3 points), naming (3 points), attention (forward digit span: 1 point, backward digit span: 1 point, vigilance: 1 point and serial 7 subtraction: 3 points), language (sentence repetition: 2 points, verbal fluency: 1 point), abstraction (2 points), delayed recall (5 points) and orientation (6 points)^1^. According to previous studies on the MoCA based on the Chinese population, we added one point to the final scores of the MoCA if the participants had 6 years of formal school-based education or fewer^16^.

### National Institute for Neurological Disorders and Stroke-Canadian Stroke Network 5-minute protocol (NINDS-CSN 5-min protocol)

The NINDS-CSN 5-min protocol was performed by extracting three items (i.e. orientation, delayed recall and verbal fluency) from the full MoCA, which results in a total score of 12^5,6^.

### Montreal Cognitive Assessment 5-minute Protocol (MoCA 5-min protocol)

The MoCA 5-min protocol was previously developed by Wong et al.^7^. The protocol first starts with an attention task by using the first trial of the 5-word delayed recall test. Second, verbal fluency score was evaluated based on the output of the effective number of words, rather than using a single cut-off value based on the number of animal names. Third, the recall test was conducted with cues (category and multiple-choice) to understand the type of memory failure, in addition to general evaluation of spontaneous recall. The total score of MoCA 5-min protocol was thus set at 30.

The Chinese (Mandarin) version of the MoCA 5-min protocol was based on the Hong Kong version of the MoCA 5-min protocol and the MoCA Chinese (Mandarin) version-1. We requested permission for research use of the assessments through the MoCA test copyright owner Dr. Ziad Nasreddine via http://mocatest.org. Only the full MoCA was administered and the scores of the MoCA 5-min protocol and NINDS-CSN 5 min protocol were obtained by rescoring the results of this single assessment.

### Neurobehavioral Cognitive Status Examination (NCSE)

The Chinese (Mandarin) version of NCSE was used in this study^15^. This examination covers 10 sections: orientations, attention, comprehension, repetition, naming, construction, memory, calculation, similarities and judgement. All sections, except orientation and memory, contain 1 screening task and series of metric tests with a gradual increase in difficulty. The total score of the NCSE is 82. In line with prior studies, a cut-off value of 65 was used to define the cut-off for cognitive impairment in patients with stroke^17^.

### Statistical analysis

SPSS version 23.0 and MedCalc version 15.6 were used for statistical analysis. Scatter plots were drawn before performing correlation analyses. Independent *t*-tests were used to compare the baseline characteristics between patients with stroke and healthy controls. ROC curves were used to examine the ability of different assessments (the MoCA, the MoCA 5-min protocol and the NINDS-CSN 5-min protocol) to differentiate cognitive impairment identified by the NCSE (NCSE < 65 out of 82) and to differentiate patients with stroke from healthy controls. The area under the receiver operating characteristic curve (AUC) was calculated for each ROC curve to describe the general diagnostic accuracy of different assessments. The optimal cut-off value was derived for each point, whereupon both sensitivity and specificity were optimized using the maximized Youden Index. The Youden Index was calculated by the equation: sensitivity + specificity – 1^18^. A non-parametric approach proposed by DeLong et al. was used to compare different ROCs^19^. Correlations among the assessments were tested by the Pearson’s correlation analysis test. Subsequent Pearson’s correlation tests were performed to explore the relationship between each item and total scores of the MoCA 5-min protocol and the NINDS-CSN 5-min protocol, with the aim to explore the individual contribution of each item. The statistically significant level was set at *P* < 0.05.

### Ethical declarations

Our investigation was carried out according to the Declaration of Helsinki. The study accords with the ethics of the Second Affiliated Hospital of Chongqing Medical University. Written informed consent was obtained from all patients.

### Informed consent

Informed consent was obtained from all individual participants included in the study.

## Results

### Characteristics of included subjects

In total, 54 stroke patients (22 from the Rehabilitation Medicine Department in the First Affiliated Hospital of Southwest Medical University and 32 from Rehabilitation Medicine Department in the Second Affiliated Hospital of Chongqing Medical University) and 27 healthy controls (3 from Rehabilitation Medicine Department in the First Affiliated Hospital of Southwest Medical University and 24 from Rehabilitation Medicine Department in the Second Affiliated Hospital of Chongqing Medical University) were recruited in this study. 25 patients were diagnosed as ischemic stroke while 29 patients were diagnosed as hemorrhagic stroke. 27 subjects with stroke were defined as cognitively normal (NCSE ≥ 65/82) and another 27 subjects with stroke were defined as cognitive impairment (NCSE < 65/82) by the NCSE. There was no significant between-group difference in age (stroke patients *vs.* healthy controls: 54.89 ± 13.46 *vs.* 50.82 ± 11.20, *t* = 1.354, *P* = 0.180) and education years (stroke patients vs. healthy controls: 10.22 ± 4.63 *vs.* 9.78 ± 4.10, *t* = 0.423, *P* = 0.674).

### Concurrent validity by correlation

**Figure 1:**
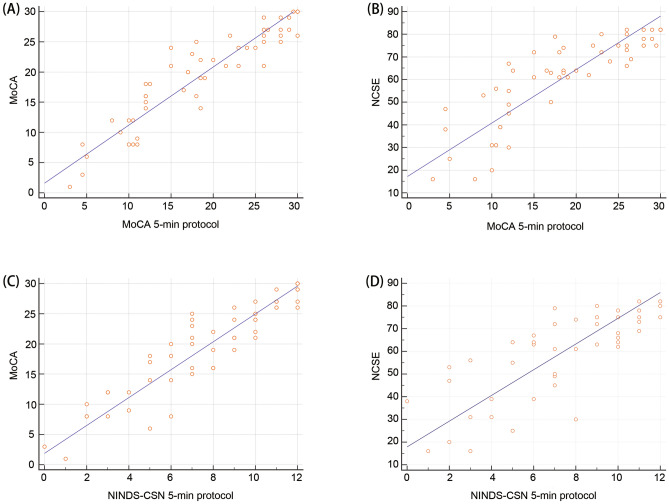
(**A**) Scatter plot of the relationship between total scores of the MoCA 5-min protocol vs the full MoCA; (**B**) Scatter plot of the relationship between total scores of the MoCA 5-min protocol and the NCSE; (**C**) Scatter plot of the relationship between total scores of the NINDS-CSN 5-min protocol and the MoCA; (**D**) Scatter plot of the relationship between total scores of the NINDS-CSN 5-min protocol and the NCSE.

Among the 54 stroke patients, subjects with sub-optimal NCSE scores (i.e. NCSE < 65) had poorer performance in the MoCA (*t* = 9.69, *P* < 0.001), the MoCA 5-min protocol (*t* = 9.11, *P* < 0.001) and the NINDS-CSN 5-min protocol (*t* = 7.57, *P* < 0.001), when compared to those within the normal range as defined by NCSE. The correlation coefficient was 0.927 when comparing the full MoCA and the MoCA 5-min protocol (*P* < 0.001), indicating that the MoCA 5-min protocol has a good concurrent validity with the full MoCA (Table 1 and Fig. 1A). Pearson’s correlation analysis between the MoCA 5-min protocol and the NCSE demonstrated a positive correlation between the two variables (*r* = 0.853, *P* < 0.001), which indicates a good concurrent validity of the MoCA 5-min protocol with the NCSE (Table 1 and Fig. 1B). Similarly, the NINDS-CSN 5-min protocol demonstrated high correlation with the full MoCA (*r* = 0.921, *P* < 0.001) and the NCSE (*r* = 0.797, *P* < 0.001) (Table 2, Fig. 1C,D). To examine the contribution from each item of the MoCA 5-min protocol and the NINDS-CSN 5-min protocol, correlation analysis between each item and total scores of the assessments was conducted. Pearson’s correlation analysis of the MoCA 5-min protocol indicated that delayed recall had strongest association with the total result (*r* = 0.897, *P* < 0.001), followed by verbal fluency (*r* = 0.858, *P* < 0.001), as well as two other items which were also associated with the total at a lesser degree (attention: *r* = 0.850, orientation: *r* = 0.625, both* P* < 0.001) (Table 1). Pearson’s correlation analysis of the NINDS-CSN 5-min protocol indicated that orientation had strongest association with the total (*r* = 0.771, *P* < 0.001), followed by delayed recall (*r* = 0.764, *P* < 0.001), and verbal fluency (*r* = 0.706, *P* < 0.001) (Table 2).

**Table 1:** Summary of correlation analyses (MoCA 5-min protocol).

	MoCA (total scores)	NCSE (total scores)	Subitems of the MoCA 5-min protocol			
			Delayed recall	Verbal fluency	Orientation	Attention
**MoCA 5-min protocol (total scores)**						
r	0.927	0.853	0.897	0.858	0.625	0.850
*P*	< 0.0001	< 0.0001	< 0.0001	< 0.0001	< 0.0001	< 0.0001

MoCA, Montreal Cognitive Assessment; MoCA 5-min Protocol, Montreal Cognitive Assessment 5-minute Protocol.

**Table 2:** Summary of correlation analyses (NINDS-CSN 5-min protocol).

	MoCA (total scores)	NCSE (total scores)	Subitems of the NINDS-CSN 5-min protocol		
			Delayed recall	Verbal fluency	Orientation
**NINDS-CSN 5-min protocol (total scores)**					
r	0.921	0.797	0.764	0.706	0.771
*P*	< 0.0001	< 0.0001	< 0.0001	< 0.0001	< 0.0001

MoCA, Montreal Cognitive Assessment.; NINDS-CSN 5-min Protocol, National Institute for Neurological Disorders and Stroke-Canadian Stroke Network 5-minute Protocol; MoCA, Montreal Cognitive Assessment.

### Concurrent validity by a known-group method

**Figure 2:**
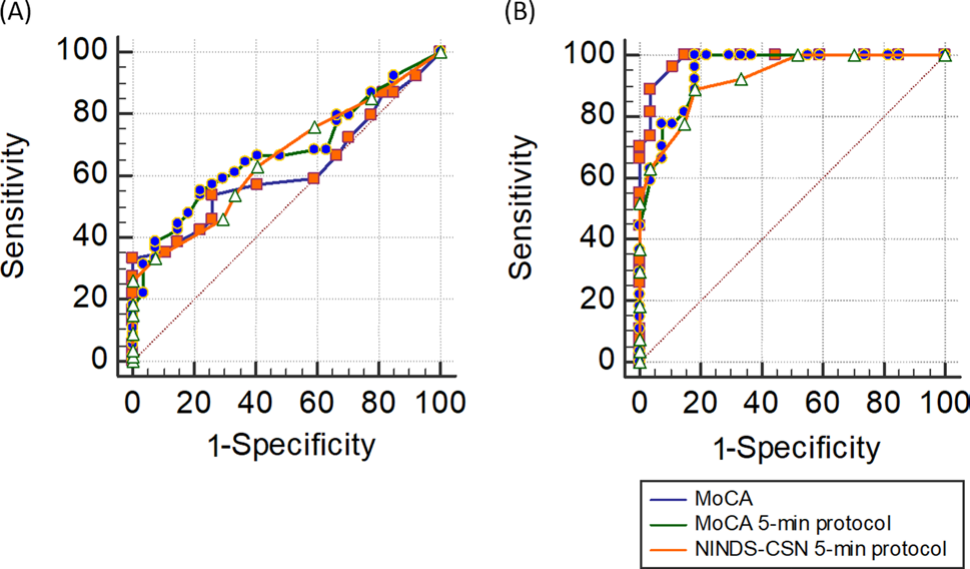
(**A**) ROC analysis (stroke patients vs. healthy controls) and (**B**) ROC analysis (stroke patients with cognitive impairments *vs.* stroke patients without cognitive impairments).

Known-groups method is another way to support concurrent validity, and we used this method to test the diagnostic accuracy of the MoCA, the MoCA 5-min protocol and the NINDS-CSN 5-min protocol in discriminating stroke patients from their healthy counterparts. We found that the MoCA 5-min protocol had the largest AUC (AUC: 0.675, 95% CI 0.562 to 0.775), followed by the NINDS-CSN 5-min protocol (AUC: 0.656, 95% CI 0.542 to 0.758), and then the full MoCA (AUC: 0.618, 95% CI 0.504 to 0.724) (Fig. 2A). However, we could not find any significant differences among the ROC curves (all *P* > 0.05).

We further tested the diagnostic accuracy of three assessments in differentiating stroke patients with cognitive impairment (NCSE < 65) from those without cognitive impairment. We found that the AUC of the MoCA 5-min protocol did not statistically differ from that of the full MoCA (AUC: 0.948 (0.851 to 0.990) *vs.* 0.984 (0.904 to 1.000), *P* = 0.0971) (Fig. 2B). The full MoCA presented with a significantly better AUC than the NINDS-CSN 5-min protocol (AUC: 0.984 (0.904 to 1.000) *vs.* 0.925 (0.819 to 0.979), *P* = 0.027). The optimal cut-off value of the MoCA 5-min protocol was 21.5, with a sensitivity of 100.0% and a specificity of 81.48%, while the optimal cut off value for the NINDS-CSN 5-min protocol was 8, with a sensitivity of 88.89% and a specificity of 81.48%. The optimal cut-off value of the full MoCA was 22, with a sensitivity of 100.0% and a specificity of 85.19%.

## Discussion

The aim of this study was to examine the concurrent validity of two short-form versions of the MoCA at cognitive screening of a cohort of stroke patients in 2 rehabilitation hospitals. We compared the two short versions of MoCA with the NCSE and found a strong association, indicating a good concurrent validity. Our study further demonstrated that the MoCA 5-min protocol, but not NINDS-CSN 5-min protocol, was comparable with the full MoCA in differentiating patients with cognitive impairment from those without cognitive impairment, when the NCSE was used as the reference criteria. The three assessments performed similarly in differentiating patients with stroke from healthy controls; however, the general diagnostic accuracy was limited.

In our study, the diagnostic accuracy of the MoCA 5-min protocol was comparable with the full MoCA according to our AUC analysis, and this finding was consistent with previous findings^4^. The AUC of the MoCA 5-min protocol was higher than the results of previous studies (AUC from 0.76 to 0.86)^4,7,12,13^, which may be because different reference standards were utilized in these studies. Previous studies have reported lower diagnostic accuracy of the NINDS-CSN 5-min protocol as compared to the full MoCA^6,9^, while the MoCA 5-min protocol performed equally well to the full MoCA^4^, which is similar to the results from our study. However, the NINDS-CSN 5-min protocol performed worse in comparison to the full MoCA, which is in line with the previous literature^20^.

Although the MoCA 5-min protocol only consists of attention tasks, delayed recall tasks, semantic fluency tasks and orientation tasks, the memory and verbal fluency tasks provided more detailed information on the patients’ cognitive functions which allows for a broader range of scores for evaluation. Impairments of the frontal-subcortical neuronal circuit have been associated with several neurocognitive impairments, including executive dysfunction and poor memory-search strategies^21^. Memory performance involves three primary processes: encoding, consolidation and retrieval^22^. Poor memory retrieval can be regarded as a subtype of executive dysfunction, which are also known as executive memory deficits^21^. If patients fail to encode new information, spontaneous recall and cued recall will also be both impaired. In contrast, if patients only have memory retrieval problems, cued recall will be preserved to a certain degree. The scoring of the memory subtask in the MoCA 5 min protocol proposed by Wong et al. was different from that in the other 2 tests. The memory subtask of the MoCA 5-min protocol required assessors to examine the patients’ performance at both spontaneous recall and cued recall, which may help to enhance the discriminative power of this protocol, as compared to the MoCA and the NINDS-CSN 5-min protocol where only the performance of spontaneous recall was assessed.

In a previous large-scale study using 1523 cases, it was reported that the verbal fluency test was comparable with the full MoCA in detecting cognitive impairments amongst the elderly population^23^. The naming fluency of the MoCA 5-min protocol is scored incrementally rather than using a single cut-off point, which makes it comparable with the full MoCA that contains the backward digit span task, clock drawing task, word similarity task and trail making task used to test executive function. Thus, with the modifications of the delayed recall task and the verbal fluency task, the MoCA 5-min protocol is likely to be a good alternative to the full MoCA as a cognitive screening tool in the Chinese stroke rehabilitation setting. Stroke patients with suboptimal assessment performance can be subjected to further detailed cognitive assessments.

Our hypothesis that the MoCA 5-min protocol was similar to full MoCA and more effective than the NINDS-CSN 5-min protocol in differentiating stroke patients from healthy controls, was not supported by the results. Also, the diagnostic accuracy of these 3 tests in differentiating stroke patients from healthy controls was not high. It is understandable that not all patients after stroke would develop cognitive impairment and some of them may not develop cognitive impairment until the chronic phase. Further study can be restricted to a stroke cohort with specific vascular lesions which are more relevant to poststroke cognitive impairment (e.g., prefrontal area) and to investigate the predictive validity of the short version of MoCA in the development of vascular mild cognitive impairment/dementia in long term.

## Limitations

Several limitations are notable in this study. First, this study was conducted in rehabilitation settings employing only the NCSE, which is a common standardized assessment used by occupational therapists, as the reference standard. Further studies will be needed to explore the concurrent validity of the short MoCA against other established neuropsychological test batteries used by neuropsychologists or even using data from clinical diagnoses conducted by neurologists. Second, we extracted the results of the short MoCA from the full MoCA directly, and we cannot exclude the possibility that recall may be delayed for a longer time with more tasks in the full MoCA, which may influence the construct validity of the short versions of MoCA. We were also unable to test the feasibility of telephone-delivered assessments in the present study. Further study will need to replicate these results for telephone administration. Third, our sample was taken from a cohort of subacute inpatients with stroke in 2 rehabilitation centers, and the generalizability to acute stroke or chronic stroke samples may be limited.

## Conclusion

The Chinese version of the MoCA 5-min protocol is a valid cognitive screening tool for identifying cognitive impairments in patients with stroke.

## Data Availability

The datasets generated and/or analysed during the current study are available from the corresponding author on reasonable request.
